# The Russianizing of the Medical Profession by the Political Machine of the A. M. A.

**Published:** 1909-10

**Authors:** G. Frank Lydston

**Affiliations:** Professor of Genito-Urinary Surgery in the State University of Illinois, Medical Department; Chicago


### TEXAS MEDICAL JOURNAL.

Established July, 1885.

F. E. DANIEL, M. D., - - - - Editor, Publisher and Proprietor.

PUBLISHED MONTHLY.—SUBSCRIPTION $1.00 A YEAR.

VOL. XXV. AUSTIN, OCTOBER, 1909. No. 4.

The publisher is not responsible for the views of contributors.

### Original Articles.

For Texas Medical Journal.

### The Russianizing of the Medical Profession by the Political Machine of the A. M. A.

BY G. FRANK LYDSTON, M. D.,

Professor of Genito-Urinary Surgery in the State University of Illinois, Medical Department; Chicago.

The principles and policies of the medical profession of America unquestionably should be officially represented by its medical societies. In times past the independent medical society and the independent journal were powerful and valuable factors in the professional life of this country. Thanks to peculiar and despotic organization methods, the independent journal and the independent society are rapidly being eliminated, and the rank and file of the profession are destined in the near future to lose all means of expression and interchange of ideas—medical and medico-political. So far as media of expression are concerned, it will be a case of the marrowless machine organs or nothing, and as these organs will be reserved for machine men and their satellites and sycophants, heaven help the outsider. Possibly, however, media of expression will not be necessary in the future. Our thinking will be done for us by the machine editors and the chosen few who are satellites of the machine.

Under the specious and hypocritic plea of unification of the profession, a few selfish and ambitious men, with the assistance of a minority of misled members of the profession at large, have constructed a political machine which has Russianized American medicine and bids fair to wipe Americanism off the medical map.

The fairer the mask which duplicity wears; the more beneficent the alleged object of wily politicians, the more dangerous the wily ones become. Especially was this so in the matter of "reorganization" of the A. M. A.

The cry of "reorganization" raised in 1901 by the coterie of medical politicians whose fingers itched for power in the A. M. A. was so speciously fair that it is not surprising that the profession at large was deceived by it or that medical men tumbled over each other in the mad rush to deliver themselves body and soul to the political bosses. A "great Journal and a great Association" had a ring which was pleasant to the ear of the unwary. Grandma's bonnet completely hid the wolf from the innocent eyes of the medical Little Red Riding Hoods. It is only of late that the rank and file have begun to realize that they were handed a gold brick in 1902—few of them really know, even now, exactly what happened at that time.

To one who knows the splendid personnel and traditions of the old organization the methods, policies and much of the personnel of the new A. M. A. are almost incomprehensible. To one who recalls the ethical principles that once pervaded the A. M. A. the history of its reorganization and the monopolistic trust to which it has given birth is almost incredible.

The professional record of our Secretary General-Editor-Manager—the chief factor in reorganization—reads like a yellow romance, and sets at naught all the principles which we are wont to inculcate in the minds of young physicians. His career shows what a lack of ethical professional principles, money-and-power-mania and inordinate ambition will do in a land where brains, talent and unblemished professional standing are supposed to be essentials of success in medicine. His present multiple powerful positions and the support he has received from shining lights in the profession put the stamp of hypocrisy on all our ethical pretensions. We have perpetrated a fraud on the high-minded young men of the profession of whom we have demanded ethical conduct. We have shattered our ideals and are wallowing in the mire of politics and commercialism. Ethics? There is no longer in medicine such a thing as ethics. "Business is business."

About 1880, a gentleman of alien birth landed in Lincoln, Nebraska. According to his own account, he went to Lincoln "to enter the State University." He evidently had agrarian notions in his head, for he matriculated in the Agricultural Department, where he remained for a year. Having learned to run a machine and to make hay while the sun shone, he abandoned his first love and resolved to become a doctor. He matriculated at a noted "we make doctors while you wait" homeopathic school—since reformed—from which he graduated in 1882.

In 1882 our homeopathic friend, with years of experience behind him—in some previous life—wrote voluminously on homeopathy, and venomously assailed "allopathy," as he termed the regular school. Straight homeopathy failing to bring rewards commensurate with his genius, our ambitious friend joined forces with the "discoverer" of the "Garten Ter-Chloride of Gold Remedy" for the alcohol and opium habits and opened an "Institute" for the cure of the sick, announcing himself in the newspapers as a specialist in three distinct branches of medicine. This "Institute" was supplied with all the known and some unknown methods of treatment of the sick—the latter including "Compound Oxygen," the *ultima Thule* of quackery in those days. The "Institute" did not thrive—alas! for "Compound Oxygen"—and our homeopathic friend was soon like Othello, with occupation gone.

His next move was newspaper specializing without the Institute attachment. In one ad. he advertised himself as a specialist in urinary and rectal diseases, guaranteeing "cures." In another he announced his titles and great skill and claimed to be a specialist in diseases of women, naively stating that he would "accommodate a few lady patients" at his residence.

After some years of adventure our hero caught a glimpse of the regular clover patch and decided that there was the realm in which glory was to be gained. Having now a clientele and at least enough wealth to enable him to dispense with advertising, he forthwith selected a regular school as his prospective alma mater. He was too busy to attend this school, so he matriculated by proxy and was clever enough to deceive the faculty into believing that he had complied with the college requirements. Examinations appear to have been a matter of form in those days, and about three weeks seem to have been sufficient to enable our homeopathic phenomenon to prepare for and take the examinations. He modestly concealed his brilliancy as a student by registering from Wisconsin. He did not want the faculty to discover that he was brainy enough to master regular medicine in Chicago the whilst he was practically continuously practicing in Nebraska. About the year 1900, our ex-advertising-homeopathic regular, by some mysterious dispensation of providence was made Editor of the *Journal* of the A. M. A., thereby falling into the softest berth he had ever occupied, one the possibilities of which he was quick to grasp. Realizing that "man that is born of woman is of few days and full of trouble," and that the head which wears a crown is not always securely fastened to the shoulders of its owner, our Editor devised a scheme worthy of a Machiavelli, whereby to give himself power, place and salary forevermore. He called to his aid a clever politician from the Southland, and formulated a plan of "reorganization" of the A. M. A. and a machine of his own which promised to keep him in the saddle for life. A new constitution and by-laws was necessary. In 1901 our two worthies succeeded in getting themselves appointed a committee on reorganization. They then wrote the document to suit themselves and for themselves. This document provided several offices for the Editor and gave him as Secretary unlimited power. This will surprise the rank and file, most of whom do not know that Drs. Simmons and McCormack, with one other man—used for padding—constituted the committee which wrote the present constitution and by-laws of the A. M. A.!

The constitution once formulated, was "put over the plate" regardless of all parliamentary and legal rules and adopted by the machine with a hurrah. A few smug, well-fed, well-paid bellwethers were all that was necessary to drive us into the medico-political shambles. When business and politics came into the door, political fairness, democracy and our time-honored ethics flew out of the window.

Thus began the Russianizing of the profession. The Secretary-General Editor-Manager has used his power to aid his friends and crush those who have opposed him. He has partially and unfairly administered his duties as Editor, using the *Journal* to abuse those whose policies displeased him, and to boom his favorites of the machine, who always have entree to the Association "organ." He has published offensive, aye, and some say, even obscene, ads. without rebuke from the Trustees. He has arbitrarily expelled at least one worthy member of the A. M. A. for opposing "reorganization" in that member's own State. He has formed subsidiary machines in various States and inspired them with contempt for legal and parliamentary obstructions to his scheme of Russianizing the Association. He has endangered the financial interests of the A. M. A. by libelous attacks in the *Journal* upon various individuals. He has absolutely dictated the policies and politics of the A. M. A. He has abused, villified and attacked the business and professional interests of everybody who has opposed him. In short he has been absolute Czar of the A. M. A. for nearly a decade and has converted our once noble Association into a despotism.

Why has he been permitted to do this? Simply because until recently no one has had the courage to attack the mighty Editor of a great medical journal and Secretary of a great Association the machine in control of which revolves around himself and will stand for anything so long as the A. M. A. is growing in membership and political power and is making money. "The king can do no wrong," and money is the god which the oligarchy of the A. M. A. asks us to worship. Most cogent reason of all is the fact that the Czar of our medical Russia dominates the politics of the Association, and practically elects the official appendages of his little machine. He owns the House of Delegates and elects his own "employers." Then, too, there is the fact that the members of the A. M. A. are with difficulty reached save through the columns of the *Journal*. These being closed to all criticism of the personnel and policies of the Association, and the majority of the independent (sic) journals being frightened to death by the Octopus, most of the avenues of protest are closed. Only by pamphleteering can the members be reached, and until I entered the arena of combat no one cared to assume the labor and expense necessary to reach the rank and file.

The machine has not been unmindful of the danger of opposition, and whenever a finger of cloud has arisen on the Association horizon the opposing element has been foregathered and conciliated by some little sop to the Cerberus of discontent. Jones, of San Francisco, for example, was once a strenuous howler for reform in the A. M. A. As soon as his voice was heard east of the Sierras he was made a Trustee, since which time he has been an ardent champion of the machine. He even goes so far as to ridicule in the columns of the State journal which he edits, members of the House of Delegates who have the temerity to oppose the machine. Jones, by the way, is the imbecile who wrote in a recent editorial in the *California State Journal,* a statement that the recent pictorial write up of two celebrated surgeons and their work, published in a secular magazine, was published and paid for by the proprietary medicine men for the purpose of discrediting the A. M. A. machine. Why Jones persists in his raucous braying, thus calling attention to the pair of long ears which flaunt so bravely over the editorial fence of the *California State Journal* is a mystery.

The fundamental requirement for membership in the A. M. A. has been a powerful weapon in the hands of the A. M. A. political machine. The provision that one must be a member of his local society in order to belong to his State organization, and the demand that he should belong to both in order to qualify for membership in the A. M. A., is the corner-stone of our medical despotism. If, my brother, you do not believe this to be dangerous, reflect on the advice which the walking delegate of the A. M. A., Dr. McCormack, publicly gives to the laity. He tells them not to employ any physician who is not a member of the local branch of the A. M. A.! Think of it! And this is America! And McCormack is the man who, with the co-operation of his charming, refined and intelligent son, who edits the *Kentucky State Journal,* is the boss of the profession of his State. *He and his son are also the men who were arrested on a State warrant for violating the health regulations of Kentucky and who were pardoned by the Governor before the case came to trial.* McCormack the younger is the man who recently stated in his editorial columns that urology is the "filthiest of specialties."

How long, think you, would present conditions endure if the members at large had a true conception of proper Association government? It is obvious that "those are best governed who are least governed." Concentration in government is always pernicious and intolerable, for power is always corruptive. All representation should be direct. Nominations for office should be by the membership and not by a machine. The records and views of all nominees should be known and freely discussed.

Have the politics of the A. M. A. subserved this principle? Supposing the last election of a Secretary had been open and by ballot, would the present incumbent have been selected? Not in a thousand years.

It requires no great power of discernment to see what will happen if the present despotism of the A. M. A. goes on unchecked. Positions of honor and trust in the A. M. A. are even now dispensed by the central gang, while positions of honor and trust in the subsidiary societies are dispensed by the State and local gangs, which are to the A. M. A. as are the tentacles of the octopus to its malevolent body. As to the outlook: Medical positions under the United States government—controlled by the gang. Medical appointments under the State—controlled by the gang. Medical offices in city or county—dispensed by the gang. Number of medical colleges—controlled by the gang. Personnel of medical faculties—dictated by the gang. Medicines which we may prescribe—ordered by the gang. Journals which shall be published—controlled by the gang. Organizations which we may join—controlled by the gang. Local health boards—controlled by the gang. "Thinks" which we shall think—controlled by the gang. Whether the gang will ever prescribe the habiliments we may wear, deponent sayeth not, but the rank and file will ever be like Alice in Wonderland, complaining of "cake yesterday and cake tomorrow, but none today."

Perhaps, after all, the chief reason for the arrogance and monopolistic trend of the machine which now governs the A. M. A. is the lack of competition. In my opinion the obligarchy will never be brought to its senses until a new and truly American Association is formed. Personally, I despair of reform in the A. M. A. until a competing association organized along democratic lines has forced the issue. I fear it would take many, many years to bring about the changes that are absolutely necessary to the reformation and purification of the A. M. A. The list of necessary reforms is somewhat appalling. As I see them they are as follows, viz.:

1. The constitution and by-laws should be amended so that the reins of power will be taken from the hands of the Secretary.

2. The offices of Manager, Secretary and Editor should be separated.

3. These offices should be filled by men of clean professional records who are not creatures of the present machine.

4. The personnel of the Board of Trustees should be almost entirely changed. The present incumbents are nearly all merely wheels and cogs in the machine.

5. The number of Trustees should be increased. The A. M. A. has outgrown the present board.

6. There should be at least three local Trustees. At present there is but one, whose chief function is to endorse the plans and actions of the Secretary General-Editor-Manager.

7. The Trustees and other officers should be selected by ballot of the members in attendance. The nominations should be made on the first day and the voting done at the place of registration on the succeeding days.

8. Provision should be made for a limited number of nominations for each office by petition.

9. Bonds should be provided for all officers upon whose shoulders rests financial responsibility. At present only the Treasurer is under bond.

10. Full itemized accounts of our business and financial affairs should be rendered the members yearly. The machine of the A. M. A. is power drunk and money mad, and sooner or later the notorious insurance scandals are likely to be duplicated by something nearer home. Human nature, be it essentially corrupt or primarily chaste as ice and pure as snow, requires a check system. We have established a "kingdom of the dollar" that is all our own, and in that kingdom hungry-eyed Graft sooner or later will crowd himself onto the throne and sit beside Ambition. *Remember that we have a business of about $500,000 per year and an expense account of nearly $400,000.*

11. A membership committee should be appointed, to hold office only one year. At present the Secretary-General is here absolute dictator.

12. A certain amount of space in the columns of the *Journal* should be set apart for free criticism, queries and comments by the rank and file. Criticism of the policies and methods of the Association should be especially invited.

13. The Editor should be a man who is capable of writing editorials. At present we are expending large sums on special editorial work. The Editor should not be the business manager of the *Journal,* but a cultured, scholarly, scientific regular physician who devotes his time solely to editorial work. Under proper conditions and restrictions and a suitably reasonable salary the present Editor might make a good business manager. I have no objection to him in a lay business capacity, in which he would not obtrude himself into the ethical, political, editorial or professional limelight. Exeunt Czar and enter employe. Speed the day!

14. The initiative and referendum should be adopted as a protection for the suffrages of the members at large.

15. Provision should be made for fairness in elections. They should be so arranged that no less than two candidates would be nominated for each office. This would in future prevent the choking off of nominations and the machine selection of officers.

16. No member should be expelled without a fair trial and a full hearing, the proceedings being published *verbatim ad literatim* in the columns of the *Journal.* This plan will obviate such damnable outrages as that perpetrated in 1904 and 1905 by the Secretary and his henchman upon Dr. Henry B. Young, of Burlington, Iowa. Any men who can in cold blood read the history of this case is unworthy of American citizenship. I regret that the Secretary did not have me to deal with at that time instead of Dr. Young, who succumbed to what he believed to be the inevitable instead of taking the matter into the courts and trying to reach the profession by pamphleteering. If any one doubts that Dr. Young —who, by the way had been President of the Iowa State Society, and for twenty-three years an honored member of the A. M. A.— was summarily expelled from the A. M. A. by the Secretary-General because he had "opposed reorganization in Iowa," let him write to the victim of the Secretary's malevolence. As for myself, I have the documentary proofs before me at the present writing. It would have been hard to have been Russianized by a regular of unblemished professional record, but to be expelled by a man who was an advertising quack and a homeopath to boot during some of the many years when Dr. Young was a respected member of the A. M. A. and whose "regular" degree was dishonestly acquired, was too much, far too much.

17. The constitution should provide that no person holding an office of trust in the Association shall be eligible to serve as a member of the House of Delegates. *If any one doubts the assertion that the A. M. A. is un-American in its present operations, let him note the porcine spectacle of the Treasurer and Trustees of the A. M. A.—men holding offices of financial trust and responsibility—serving as delegates to the body which elects them and voting for themselves.* They also serve as electors in the State societies and vote for themselves for Delegates to the A. M. A. Great system, isn't it? Then let the skeptic refer to the Constitution of the United States, which prohibits any person holding an office of trust under the government from election to the office of Senator or Representative or serving as a member of the Electoral College. It also prohibits a Senator or Representative from serving as an Elector. In the A. M. A. a man may serve as Treasurer, or Trustees, Delegate and Elector.

No person holding an office of trust in the A. M. A. or State society should serve in the State House of Delegates.

In passing let me call attention to the fact that the only answer thus far made to my various charges against the record of the Secretary General-Editor-Manager has been what is termed in law a "plea of confession and avoidance." Mud has been thrown at me by the oligarchy, but the profession of this country knows my record and my attitude toward fakers and crooked politics too well to do aught but laugh at the puerilities and falsehoods of the machine politicians who are running the A. M. A. Frankly, I don't blame the cornered rat who shows his teeth. Nature is strong within him. The machine politicians who are fighting so desperately the battles of the ex-quack and irregularly made "regular" who leads them, are fighting for their political lives. And they are fighting vainly. *Only a little while longer, my brethren. The rank and file are waking from their sleeping sickness.*

